# Optimization and Characterization of Bioactive Metabolites from Cave-Derived *Rhodococcus jialingiae* C1

**DOI:** 10.3390/biom15081071

**Published:** 2025-07-24

**Authors:** Muhammad Rafiq, Umaira Bugti, Muhammad Hayat, Wasim Sajjad, Imran Ali Sani, Nazeer Ahmed, Noor Hassan, Yanyan Wang, Yingqian Kang

**Affiliations:** 1 Guizhou Key Laboratory of Microbiome and Infectious Disease Prevention and Control, Key Laboratory of Environmental Pollution Monitoring and Disease Control, Ministry of Education of Guizhou, School of basic Medical Sciences, Joint Laboratory of Shanghai Dongli One Health Research Institute Co., Ltd., Guizhou Medical University One Health Research Institute, Gui’an 561113, China; mr14311@my.bristol.ac.uk (M.R.);; 2 Department of Microbiology, Faculty of Life Sciences and Informatics, Balochistan University of IT, Engineering and Management Sciences, Quetta 87300, Pakistan; 3 State Key Laboratory of Microbial Technology, Institute of Microbial Technology, Shandong University, Qingdao 266237, China; hayatqau89@gmail.com; 4 Key Laboratory of Cryospheric Science and Frozen Soil Engineering, Northwest Institute of Eco-Environment and Resources, Chinese Academy of Sciences, Lanzhou 730000, China; 5 Department of Biotechnology, Faculty of Life Sciences and Informatics, Balochistan University of IT, Engineering and Management Sciences, Quetta 75000, Pakistan; 6 National Institute for Biotechnology and Genetic Engineering-College (NIBGE-C), Pakistan Institute of Engineering and Applied Sciences (PIEAS), Faisalabad 39000, Pakistan

**Keywords:** extremotolerant bacteria, caves microbes, novel bioactive metabolites, antibiotics

## Abstract

Extremophilic microorganisms offer an untapped potential for producing unique bioactive metabolites with therapeutic applications. In the current study, bacterial isolates were obtained from samples collected from Chamalang cave located in Kohlu District, Balochistan, Pakistan. The cave-derived isolate C1 (*Rhodococcus jialingiae*) exhibits prominent antibacterial activity against multidrug-resistant pathogens (MDR), including *Escherichia coli*, *Staphylococcus aureus*, and *Micrococcus luteus*. It also demonstrates substantial antioxidant activity, with 71% and 58.39% DPPH radical scavenging. Optimization of physicochemical conditions, such as media, pH, temperature, and nitrogen and carbon sources and concentrations substantially enhanced both biomass and metabolite yields. Optimal conditions comprise specialized media, a pH of 7, a temperature of 30 °C, peptone (1.0 g/L) as the nitrogen source, and glucose (0.5 g/L) as the carbon source. HPLC and QTOF-MS analyses uncovered numerous metabolites, including a phenolic compound, 2-[(E)-3-hydroxy-3-(4-methoxyphenyl) prop-2-enoyl]-4-methoxyphenolate, Streptolactam C, Puromycin, and a putative aromatic polyketide highlighting the C1 isolate chemical. Remarkably, one compound (C_14_H_36_N_7_) demonstrated a special molecular profile, signifying structural novelty and warranting further characterization by techniques such as ^1^H and ^13^C NMR. These findings highlight the biotechnological capacity of the C1 isolate as a source of novel antimicrobials and antioxidants, linking environmental adaptation to metabolic potential and supporting natural product discovery pipelines against antibiotic resistance.

## 1. Introduction

Antimicrobial resistance (AMR) is a serious threat to contemporary medicine, compromising the success of organ transplantation, surgery and chemotherapy. It has been estimated that about 70,000 annual deaths are mainly due to infections caused by drug-resistant pathogens, which can be projected to rise to ten million by 2050 if the AMR issue is not appropriately addressed [1]. The AMR arises mainly due to the environmental transmission of AMR genes, improper waste management, and the improper use of antibiotics in both agricultural and clinical settings [2,3,4]. The SARS-COV-2 pandemic exacerbated this crisis by promoting widespread antibiotic co-treatment and increasing secondary infections caused by multidrug-resistant bacteria and fungi, predominantly in immunocompromised or hospitalized patients [1,5,6,7,8,9,10]. This is projected to contribute to the persistent emergence of AMR [11,12,13,14,15].

Although antimicrobial resistance looms as a formidable public health crisis, the antibiotic innovation remains stalled. Currently, among the 30–40 antibacterial candidates in clinical trials targeting the WHO priority pathogens, many belong to the known classes of antibiotics. Notably, only a small fraction (~25%) of antibiotics in clinical trials belong to a novel class or possess unique mechanisms of action [16,17], although none of these exhibits antimicrobial activity against the WHO priority pathogens or the ESKAPE pathogens. This gap underscores the need to explore novel sources of bioactive compounds.

Traditionally, natural products (NPs) have remained a cornerstone in the discovery of anti-infective agents. A significant portion of modern therapeutics, accounting for nearly 75% of current anticancer and 69% of anti-infective agents, are either natural products or their derivatives [18,19]. Their structural complexity, including higher rigidity, molecular weight, and hydrogen-bonding ability, enables natural products to occupy a broader chemical space, which modulates complex biological interactions compared to synthetic compounds [20,21,22]. However, issues such as high rediscovery and challenges in screening and isolation have abridged the pharmaceutical industry’s investment in NP-based programs. Nevertheless, current advancements in genome mining and metabolomics have revitalized scientific curiosity in microbial natural products, particularly from previously untapped ecological niches [23].

Natural products are produced by almost all kinds of organisms, including plants, fungi, algae, and bacteria found in diverse habitats [24], such as deserts, mud volcanoes, glaciers, and caves, which possess unique metabolic abilities, including novel chemical scaffolds with therapeutic potential.

Among these environments, one prominent habitat is the cave, characterized by low light intensity, temperature, and nutrients, and high humidity [25]. These inimitable features might encourage the production of bioactive compounds in cave microorganisms [26].

Keeping in mind the importance of cave bacteria, this study was designed to investigate natural products with potent antibacterial and antioxidant activities derived from a cave-derived C1 isolate (*Rhodococcus jialingiae*), offering new avenues to address the global AMR crisis.

## 2. Materials and Methods

### 2.1. Study Area

**Figure 1:**
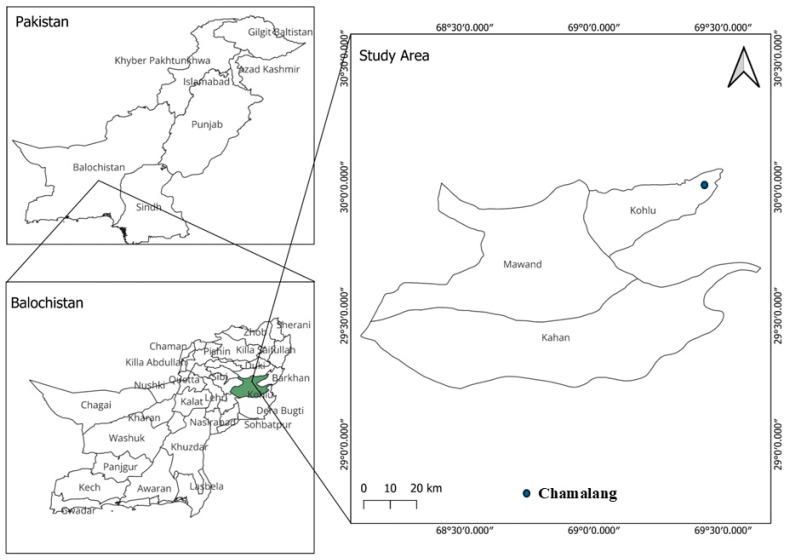
Geological map of Pakistan indicating the location of the sampling sites Chamalang, Kohlu, Balochistan.

Soil samples were collected from different zones of the Chamalang Cave in Kohlu District, Balochistan, Pakistan. To maintain sterility, the samples were collected in UV-treated Falcon tubes. Samples were transported to the Department of Microbiology at BUITEMS, Quetta, and stored at 4 °C for further analysis [27] (Figure 1).

### 2.2. Isolation and Characterization of Bacteria

The bacterial isolation and identification were carried out according to [28]. Briefly, samples were serially diluted, and then 100 µL of the most diluted sample was spread on Luria–Bertani (LB) agar plates and incubated at 37 °C for 3 to 7 days. Colonies with distinct pigments and morphologies were selected and further investigated for their antimicrobial activities, characterized through Gram reaction, physiological and phenotypic properties, and 16S rRNA gene sequence. The isolates were reliably cultured on LB agar and stored as glycerol stocks at −80 °C.

### 2.3. Screening for Antimicrobials Production

All isolates were screened against reference pathogenic strains (*E. coli* ATCC 25922, *P. aeruginosa* ATCC 27853, *S. aureus* ATCC 25923, and *B. subtilis* ATCC 6633) using the agar well diffusion method. Briefly, the bacterial suspensions were prepared according to the 0.5 McFarland standard and spread uniformly on Mueller–Hinton Agar (MHA). Wells were prepared with a sterile borer, sealed with molten MHA, and filled with crude bacterial extracts. They were then incubated for 24 to 48 h at 37 °C. The zone of inhibition was measured as previously described by [29]. The isolate C1 was selected for further processing based on its potential for increased antibiotic production.

### 2.4. Optimization of Physicochemical Conditions

Biomass and metabolite yields require an optimal nutrient source, concentration, as well as specific abiotic factors such as pH, temperature, and agitation. To enhance biomass and secondary metabolite yield, growth parameters, including media, pH, temperature, carbon and nitrogen source, and concentration, were optimized as described earlier [30,31,32]. The optimization parameters for bioactive compound production are given in Supplementary Table S1.

### 2.5. Molecular Identification of the Isolate

Genomic DNA from the C1 isolate was extracted using the NucleoSpin Microbial DNA isolation kit, designed by Takara Inc., Shiga, Japan. To determine the taxonomic identity of the isolate, the 16S rRNA sequence was amplified using primer 27F/1492R (5′AGAGTTTGATCMTGGCTCAG3′/5′TACGGYTACCTTGTTACGACTT3′). The amplified sequence was further confirmed by agarose gel electrophoresis using the Takara wild-type marker (100–5000 base pairs). Further identification of the amplified DNA was confirmed using Sequencing (Macrogen, Inc., Seoul, Republic of Korea).

### 2.6. Phylogenetic Analysis

The resulting sequence was analyzed using the BLAST search tool (www.ncbi.nlm.nih.gov/blast) to identify the isolate through an evaluation of the NCBI database. The strain C1 was found to be 99.65% similar to *Rhodococcus* species. The relative sequences were obtained from the NCBI database, and the phylogenetic tree was constructed using MEGA X 10.0.4 software. The sequence was submitted to the NCBI GenBank database under the accession PV565619.

### 2.7. Biochemical Characterization of C1 Strain

To further characterize species-level differences among *Rhodococcus* species, several biochemical tests, including Tween-80 hydrolysis, gelatin liquefaction, and starch hydrolysis assays, were evaluated for enzymatic activity. These tests were performed using established microbiological protocols, and the biochemical tests were assessed based on visible changes in the corresponding media after incubation. The results were noted for comparative analysis to confirm the species of the C1 isolate.

### 2.8. Scanning Electron Microscopy (SEM) of C1 Strain

Scanning electron microscopy (SEM) was performed to study the detailed cell morphology of the C1 isolate. Bacterial cells were fixed, dehydrated through a graded ethanol series, and sputter-coated with gold to improve conductivity. SEM images were captured at 40,000× magnification with a 5 µm scale bar.

### 2.9. Lab-Scale Fermentation Using Optimized Specialized Medium (SM)

Approximately 30 L of fermentation was carried out in 300 mL Erlenmeyer flasks with 150 mL of Optimized Specialized Medium (SM), a modified form of specialized media (peptone 1.5 g/L, MgSO4 2 g/L, NaCl 3 g/L, KCl 2 g/L, Tween 80 1.8%, and glycerol 0.3% *v*/*v*). The media was designed after systematic optimization of the isolate C1, which contained additional peptone and glucose at 1.0 and 0.5 g/L, respectively, as the optimum nitrogen and carbon sources, along with the addition of trace minerals. The pH was adjusted to 7.0 (Supplementary Table S2). The medium containing 1% of the seed culture of C1 was incubated at 30 °C with agitation at 180 rpm for 7 days, which are optimal conditions for both biomass and extractable compounds.

### 2.10. Extraction of Natural Products

After incubation, the broth culture was centrifuged at 4 °C and 10,000 rpm for 10 min. The supernatant was collected in a specially designed flask. The compounds were then extracted with ethyl acetate at a partitioning ratio of 1:3. The ethyl acetate fraction was subsequently subjected to rotary evaporation to concentrate the crude extract.

### 2.11. Compound Fractionation

#### Flash Column Chromatography

The crude extract of C1 isolate was further processed to make fractions based on polarity. The Flash column was packed with silica gel (mesh size 60–120). Before carefully loading the sample, the column was equilibrated with methanol. Elution was carried out using a stepwise gradient of methanol, ranging from 20 to 100%, at a flow rate of 10 mL/min. The obtained fractions were subjected to rotary evaporation to make concentrated fractions. A total of 5 fractions were collected from Flash column chromatography.

### 2.12. Bioassays of the Crude Fractions

#### 2.12.1. Antibacterial Activity

The antibacterial activity of all five fractions was evaluated against ATCC strains, encompassing both Gram-negative and Gram-positive bacteria including *Micrococcus luteus*, *Escherichia coli*, *Staphylococcus aureus*, *Enterococcus faecium*, and *Bacillus thuringiensis*. A bacterial suspension was prepared according to the 0.5 McFarland standard. Sterile cotton swabs were dipped slightly into the bacterial suspensions, and a uniform lawn was prepared on Mueller–Hinton Agar (MHA) plates.

The disc diffusion method was employed to evaluate antibacterial activity. Sterile filter paper discs with a diameter of 6 mm were soaked in the specific crude extract and labeled correctly. The plates were incubated at 37 °C for 24–48 h, then the zones of inhibition were measured and recorded for each test organism.

#### 2.12.2. Antioxidant Assay

The antioxidant potential of the metabolite was assessed using the DPPH assay. A 0.2 mM solution of DPPH was prepared in methanol. Serial dilutions of the metabolite (50–350 µg/mL) were prepared. Ascorbic acid, a known antioxidant, was used as a positive control at the same concentration range (50–350 µg/mL) for comparative evaluation. A blank was prepared using methanol alone. Equal volumes (500 µL each) of the extracted metabolite and DPPH solution were mixed in a test tube and incubated in the dark for 30 min to allow the reaction to proceed. A similar procedure was adopted for ascorbic acid. Absorbance was measured at 517 nm using a UV-visible spectrophotometer. A decrease in absorbance indicated antioxidant (radical scavenging) activity. The percentage of DPPH radical scavenging activity (RSA) was calculated using the following formula:

RSA (%) = [(A_control − A_sample)/A_control] × 100

where

A_control is the absorbance of the DPPH solution without the sample.
A_sample is the absorbance of the DPPH solution containing the metabolite [33].

#### 2.12.3. Sephadex LH-20 Column Chromatography

To further evaluate the bioactive compounds, the most prominent and active fractions, F2 and F5, were subjected to Sephadex LH-20 Column Chromatography. The samples were dissolved in methanol. The gel LH-20 (hydroxypropylated dextran) was washed with methanol, and the sample was loaded uniformly onto it. The elution speed was fixed at one drop every 10 s, and 3 mL of the sample was collected.

#### 2.12.4. Thin-Layer Chromatography

TLC was performed on 6 cm silica gel plates for the qualitative analysis of the metabolites. A clear line was drawn at the baseline of the TLC plate, just above the solvent level, to prevent sample mixing. Samples were applied to the baseline using a capillary tube, allowed to air-dry, and the plates were then placed in a pre-saturated glass chamber containing a solvent system of methanol and chloroform at a specific ratio. Once the solvent front reached an appropriate height, plates were removed and dried.

### 2.13. High-Performance Liquid Chromatography

The fractions were further confirmed via HPLC. Dried fractions were dissolved in methanol and then centrifuged at 12,000 rpm for 25 min to remove any particulates. Approximately 100 µL of the clear supernatant was transferred into a clean HPLC tube for further analysis. TLC and HPLC were used to pool similar compounds and separate the distinct ones. The samples were subsequently screened for antimicrobial and antioxidant properties.

### 2.14. Isolation of the Compounds Using Reverse Phase HPLC

Bioactive fractions obtained from Sephadex LH-20 were further purified using reverse-phase high-performance liquid chromatography (RP-HPLC) equipped with a C18 column. Separation was achieved using a water–acetonitrile gradient under UV detection, allowing for the precise isolation of individual compounds. Peak fractions were collected based on retention time and purity, confirmed by analytical HPLC. The isolated compounds were then subjected to structural elucidation by QTOF-MS.

### 2.15. QTOF-MS Analysis for Compound Identification

The Quadrupole Time-of-Flight Mass Spectrometer (QTOF-MS) was calibrated using a standard calibration solution recommended by the manufacturer. The instrument was operated in both positive and negative ionization modes over a mass range of 100 to 1500 *m*/*z*. Ion source conditions were optimized for sensitivity, with a capillary voltage of ±4.5 kV (depending on the mode), a source temperature of 120 °C, and a desolvation gas flow rate of 800 L/h. Bioactive fractions were injected via an autosampler at a flow rate of 0.8 mL/min. Data acquisition was performed in full-scan mode, and MS/MS experiments were conducted on the five most abundant ions for structural elucidation. The acquired spectra were processed using specialized software to ensure data quality and consistency. Metabolite identification was revealed on accurate mass measurements, isotopic distribution, and MS/MS fragmentation patterns. These features were matched against established databases such as METLIN and MassBank.

### 2.16. Statistical Analysis

All experiments were performed in biological triplicate, and the results are reported as mean ± standard deviation. Linear regression, correlation, and 2-way ANOVA were used. All analyses were conducted in RStudio version 4.4.1, and differences were considered statistically significant at *p* < 0.05.

## 3. Results

### 3.1. Morphological and Cultural Properties of the Isolates

In the current study, two bacterial isolates with distinct pigmented colonies and morphological and cultural characteristics were selected (Supplementary Figure S1). The isolates were designated as C1 and C2, and each was categorized accordingly. The cultural characteristics and Gram reactions of these isolates are given in Supplementary Table S3.

### 3.2. Antimicrobial Screening Test

The strains were screened and tested for antibacterial activity against multidrug-resistant (MDR) strains. Among the cave-derived isolates, C1 showed extraordinary activity against all pathogenic bacteria. Two isolates showed moderate activity, while the other isolates did not exhibit any antibacterial activity. Based on its remarkable antimicrobial activity, the C1 isolate was further selected for the production of secondary metabolites. The details are given in Supplementary Figure S2.

### 3.3. Molecular and Biochemical Identification

**Figure 2:**
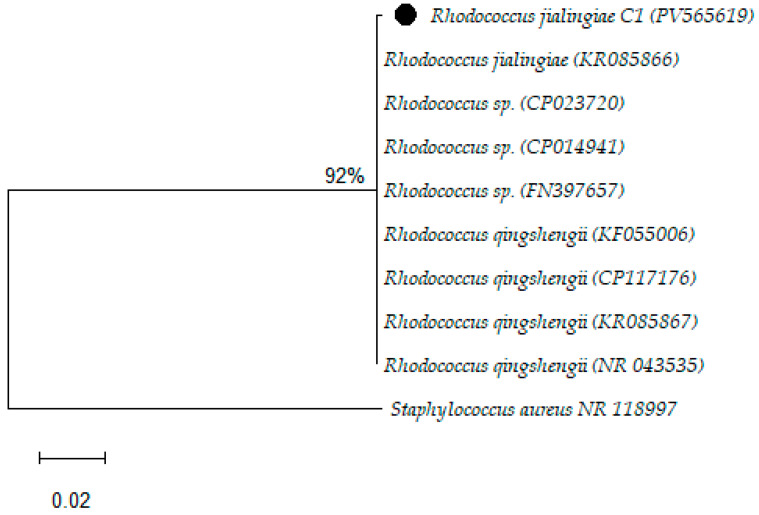
Phylogenetic analysis of the study isolates by the Maximum Likelihood method.

The BLAST search analysis of the 16S rRNA sequence revealed that the isolate C1 belongs to the family *Nocardiaceae* and the genus *Rhodococcus*. The sequence displayed a high degree of similarity (99.65%) with three closely related species: *Rhodococcus jialingiae*, *Rhodococcus qingshengii*, and *Rhodococcus erythropolis*, as described in Figure 2.

### 3.4. Biochemical Characterization

The close sequence similarity among these taxa necessitates species-level identification and biochemical characterization. Numerous enzymatic analyses, including Tween-80, lipid hydrolysis, and gelatin liquefaction, as well as amylase production, were carried out. Interestingly, the C1 isolates failed to produce any of these enzymes. Based on these results, C1 was identified as *Rhodococcus jialingiae*, as shown in Supplementary Table S4 and Supplementary Figure S3.

### 3.5. Scanning Electron Microscopy (SEM)

**Figure 3:**
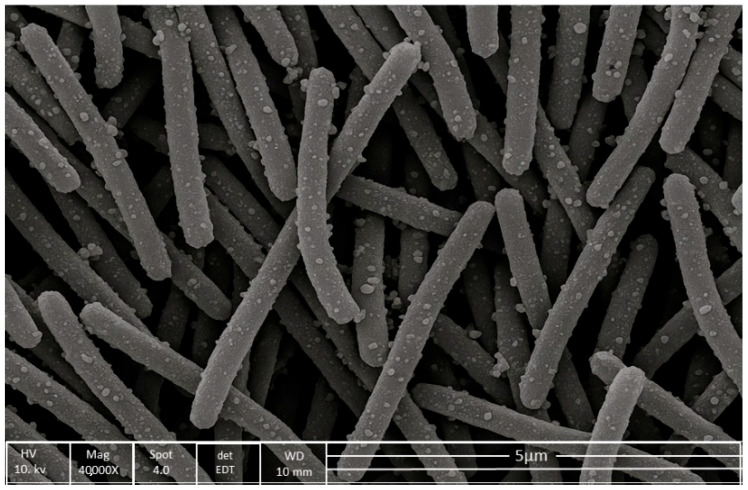
Scanning electron microscopy (SEM) image of the C1 bacterial isolate captured at 40,000× magnification and 5 µm scale. The cells are rod-shaped, measuring approximately 3–5 µm in length and 0.5–1.0 µm in width.

To study the detailed structural and morphological analysis of the C1 isolate, scanning electron microscopy was used. The cells were observed at a scale bar of 5 µm and 40,000× magnification. A 20 h old sample was used for SEM analysis, revealing bacteria as rod-shaped, ranging in length from 3 to 5 µm and in width from 0.5 to 1.0 µm. The bacterium C1 cells appear rough and granular, which could be due to the production of extracellular polymeric substances (EPS), as shown in Figure 3.

### 3.6. Optimized Physicochemical Conditions

#### 3.6.1. Hierarchical Clustering of Biomass Production over Time Across Media

Biomass production was checked at 24 h intervals for up to 192 h under four different media: Luria–Bertani broth (LB), nutrient broth (NB), specialized medium (SM), and tryptic soy broth (TSB). The subsequent data was displayed as a heatmap, demonstrating both temporal shifts and medium-dependent variability.

**Figure 4:**
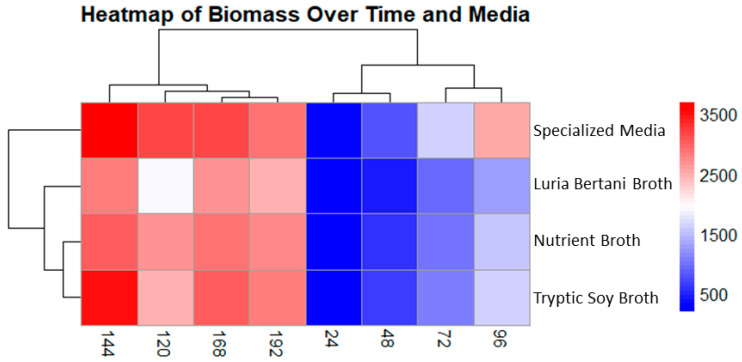
Cluster heatmap displaying biomass production in four different media. Columns show sampling time interval, and rows indicate media used. Colors indicate low (blue) and high (red) biomass concentration (µg/L). Specialized medium showed the best yield for biomass production at 144 h followed by tryptic soy broth.

Of all the tested media, SM yielded the most pronounced biomass production, reaching a maximum of 3693 µg/L at a 144 h time interval, followed by TSB, which reached a maximum of 3507 µg/L. Interestingly, LB and NB also exhibited a similar growth pattern, peaking at 2800 and 3021 µg/L, respectively. In all four media, the biomass dropped marginally after 144 h. The heatmap displayed a clear, unique grouping pattern, with SM and NB showing comparable profiles during their ultimate phases. The biomass production was visualized through a gradient ranging from blue (low) to red (high). Remarkably, the decline in biomass in all four media after 144 h suggests a mutual alteration that is likely due to nutrient scarcity or the accumulation of inhibitory substances, marking a shift in growth dynamics (Figure 4).

#### 3.6.2. Extractable Compound Production over Time Across Media

**Figure 5:**
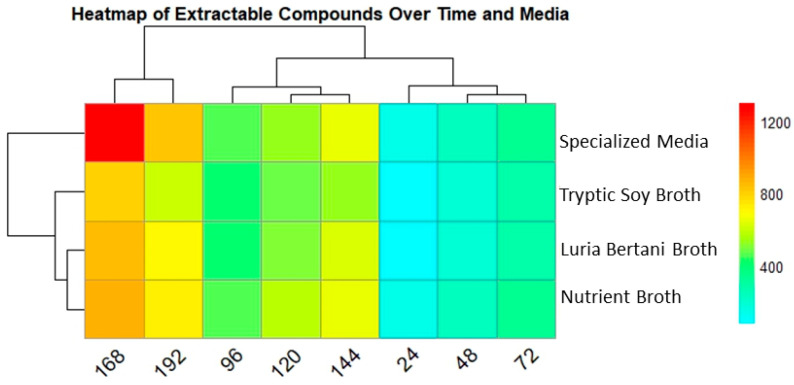
Heatmap presenting secondary metabolite production in all 4 media over time. Columns indicate time points (24–192), while rows show the media used. Color intensity specifies secondary metabolite production concentrations (µg/L), red (high) and light blue (low). SM showed the highest yield followed by TSB, after 168 h.

The extractable compound was also tracked similar to that of biomass in all four media. The heatmap displays unique medium-dependent patterns for extractable compounds. The specialized media (SM) showed the highest metabolite production, 1297 µg/L, at 168 h. Interestingly, metabolite production peaked after biomass decline, signifying a possible metabolic shift that may be due to stress adaptation or the onset of secondary metabolism. NB and LB also followed a similar pattern, with extractable compounds of 867 µg/L and 853 µg/L, respectively, at 168 h. Despite showing comparatively constant biomass, TSB exhibited lower secondary metabolite production at 793 µg/L in all time points. The clustering analysis showed that SM and NB were reliably grouped together, representing common metabolic circumstances favorable for improved metabolite production Figure 5.

#### 3.6.3. Correlation Between Biomass and Extractable Compounds Across Media

A strong positive correlation (Pearson’s r = 0.97–0.99) was revealed between biomass and extractable compounds across all media used. The SM showed the highest correlation (r = 0.99), followed by TSB and NB (r = 0.98), and LB (r = 0.97), indicating a near-linear association between biomass and extractable metabolite production, with slight variations in slope among media. Scatter plots with fitted regression lines demonstrate these trends, highlighting the potential of biomass production as a proxy for extractable metabolite yield under varying conditions (Supplementary Figure S4).

#### 3.6.4. HPLC Analysis of Metabolites Production Among Media

**Figure 6:**
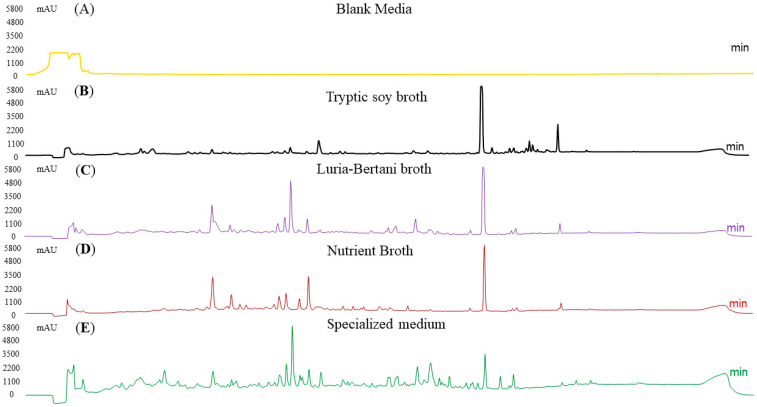
HPLC analysis of secondary metabolites production across different media at optimum conditions. SM revealed the maximum peak diversity, followed by NB and LB. Extracts from TSB had fewer peaks, representing lower metabolite production. A blank media (media with no bacteria) was also run as a control.

Four different media were used for optimization. A blank media was used as a control. To validate the extractable metabolites, we performed an HPLC analysis. Among these, SM produced the maximum metabolites, suggesting the highest chemical diversity in this media, followed by NB and LB media, though both showed a similar pattern. The TSB medium produced the fewest peaks. In summary, the culture conditions extensively influenced metabolite yields, with specialized media producing a diverse metabolite profile, followed by NB. To ensure clarity, SM without bacterial incubation was run on the HPLC to determine the baseline peaks for comparison with those from bacterial incubation (Figure 6).

### 3.7. Impact of pH on Biomass and Extractable Metabolites Production

**Figure 7:**
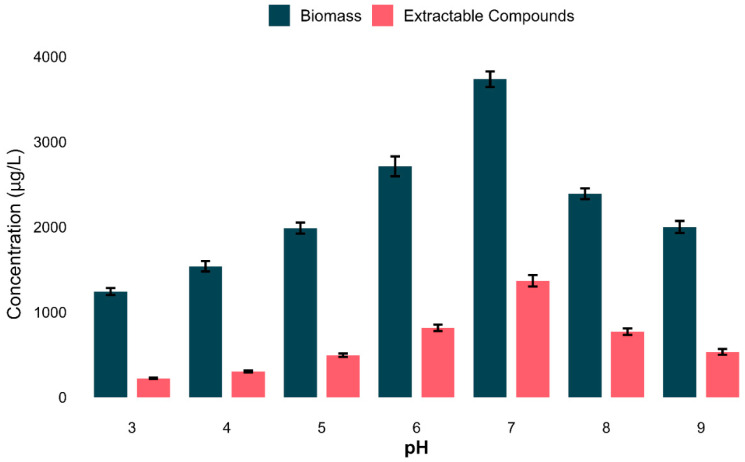
Impact of pH on bacterial growth and secondary metabolites production. Bar plot revealing the average biomass and secondary metabolites (µg/L) in all tested pHs. The optimum condition was pH 7, for both biomass and metabolite yield. Data shows t means ± standard deviation of three biological replicates.

The influence of the pH on microbial growth and extractable compound production was scientifically assessed. Both biomass and secondary metabolite production increased gradually from acidic to neutral and declined again at the basic level. The highest biomass (average: 3735.4 µg/L) and extractable compound yield (average: 1367.7 µg/L) were recorded at pH 7 (Figure 7).

To investigate the relationship between secondary metabolite yield and biomass production, a correlation analysis was conducted for all scenarios. A robust positive linear relationship (Pearson’s *r* = 0.995, *p* = 3.2 × 10^−6^) was observed, indicating that bacterial cell density substantially contributes to metabolite yield under the established parameters (Supplementary Figure S5). To further illustrate these patterns, linear regression lines were plotted across the entire pH range in Supplementary Figure S6. Both biomass and extractable compound yields showed a unimodal response at pH 7, indicating a synchronized regulation of both biomass and extractable compounds. These results establish that pH 7 was the ideal condition for both biomass and extractable metabolites.

### 3.8. Effect of Temperature on Biomass and Extract Yield

To investigate the effect of incubation temperature on both growth (biomass) and secondary metabolite production, experiments were conducted over a range of temperatures (10 to 37 °C). A strong temperature-dependent response was observed for both biomass and metabolite yields. Both biomass and metabolite yield gradually increased from 10 °C, peaking at 3442.3 ± 48.6 µg/L and 1296.3 ± 34.3 µg/L, respectively, at 30 °C. 

**Figure 8:**
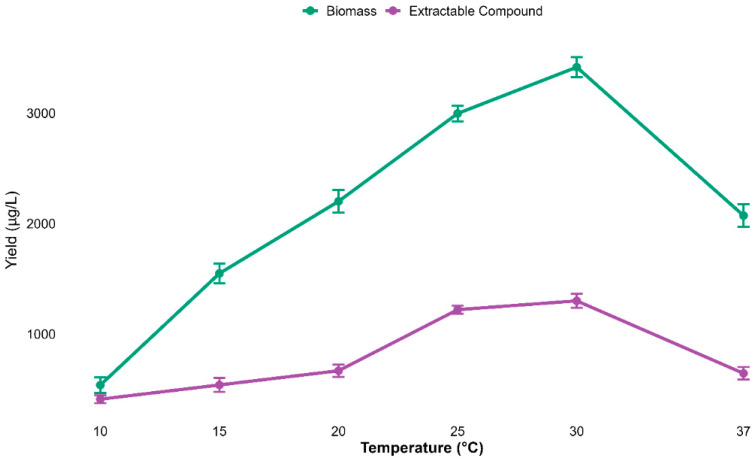
Biomass and metabolite yields increased with temperature, peaking at 30 °C and declining at 37 °C. Data are mean ± s.d. (*n* = 3), indicating 30 °C as optimal.

Collectively, the optimum temperature for both biomass and compound yield was 30 °C, though both biomass and metabolites declined expressively at 37 °C (Figure 8).

A minimal, though statistically significant, correlation was noted between biomass and extract yield at different temperatures (Pearson’s r = 0.089, *p* = 0.043), signifying only a marginal relationship between biomass and metabolite yield, as described in Supplementary Figure S7.

### 3.9. Impact of Nitrogen and Carbon Sources on Biomass and Extractable Compounds

**Figure 9:**
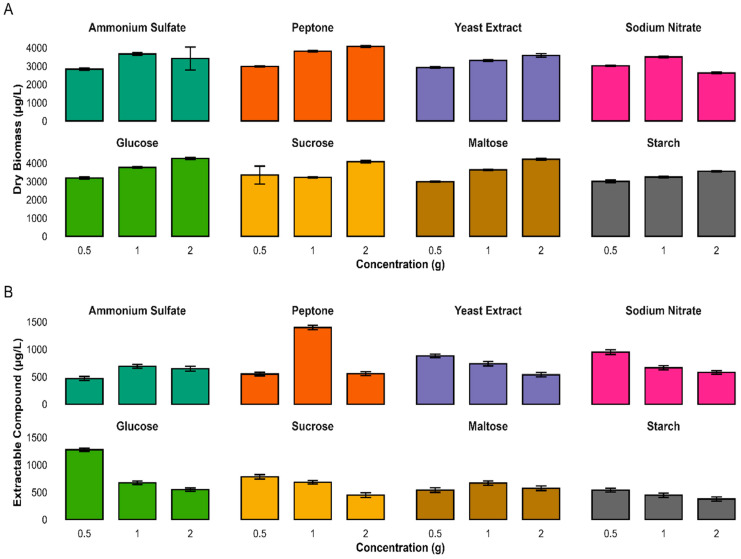
Impact of nitrogen and carbon sources and concentrations on microbial biomass and extract yield. The Bar plots show dry biomass (**A**) and extractable compounds (**B**) from cultures grown with four N and four C sources. Values are mean ± s.d. (*n* = 3). Peptone and glucose supported the highest biomass; extract yield peaked with peptone (1.0 g). Nutrient type and concentration strongly influenced growth and metabolite production.

To study whether different nitrogen (N) and carbon (C) sources have an impact onbiomass and secondary metabolite production, four different N and C sources were tested at three concentrations each(0.5, 1, and 2 g/L). Among the nitrogen sources, peptone and glucose (2 g/L) each produces the maximum biomass (4074 µg/L and 4254 µg/L) correspondingly, though lowest biomass was observed in sodium nitrate and maltose (2 g and 0.5 g/L), respectively. Similarly, peptone and glucose (1 g and 0.5 g/L) produced the maximum extractable metabolites; however, the lowest metabolites extraction was recorded for starch and sucrose (2 g/L) (Figure 9).

A 2-way ANOVA indicated that both nitrogen type and concentration significantly impacted biomass and secondary metabolite production (*p* < 0.001). The interaction between nitrogen source and concentration was statistically significant, indicating that microbial responses varied depending on the specific conditions (Supplementary Table S5).

Similarly, a two-way ANOVA of carbon source and concentration also showed statistically significant results for both biomass and extractable compound yield (*p* < 0.001). A substantial interaction effect was also observed, showing that carbon concentration varied based on the carbon source used. These analyses highlighted the importance of optimizing both variables to enhance biomass and extractable compound production (Supplementary Table S6).

### 3.10. HPLC Analysis of Optimal Nitrogen and Carbon Sources

**Figure 10:**
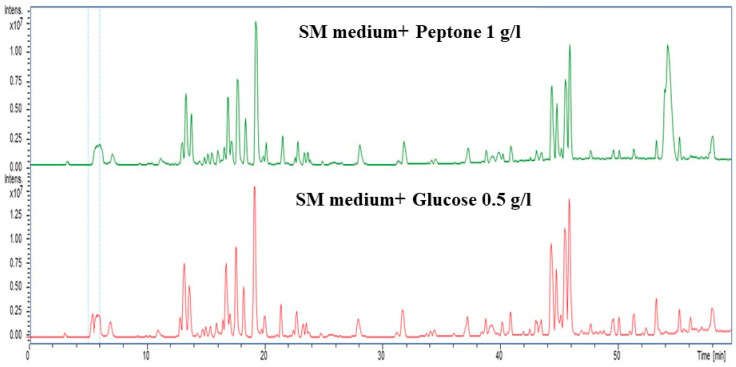
HPLC chromatograms showing distinct metabolic profiles for nitrogen (peptone, 1 g/L; green) and carbon (Glucose, 0.5 g/L; red) sources. Peptone led to the production of nitrogen-rich metabolites (20–25 and 40–45 min), while maltose produced carbohydrate-related peaks (20 and 50 min), highlighting nutrient-specific metabolic shifts.

To further confirm the impact of nitrogen and carbon source and concentration, we supplemented the SM medium with peptone (1 g/L) and glucose (0.5 g/L) to enhance extractable metabolites. The samples were then analyzed using high-performance liquid chromatography (HPLC) analysis. Both peptone and glucose exhibited distinct peaks, indicating that at such low concentrations of both N and C sources, secondary metabolites were enhanced. These contrasting chromatograms indicate that the nitrogen and carbon sources markedly shape the secondary metabolism (Figure 10).

### 3.11. Lab-Scale Fermentation

Based on these analyses, the optimum conditions, including specialised media, pH 7, a temperature of 30 °C, peptone at 1 g/L, and a carbon source of glucose at 0.5 g/L, were adjusted for lab-scale fermentation. A total of 30 L of fermentation was carried out in a 300 mL flask containing 150 mL of specialized media, modified according to our experiment. After fermenting for 7 days, the crude compound was extracted using 3× ethyl acetate. The samples were evaporated using a rotary evaporator, and approximately 8 g of the compound revealed a recovery efficiency of 267 mg/L.

### 3.12. Compound Purification and Antibacterial

The crude extract was fractionated by Flash chromatography using silica gel and a gradient of methanol ranging from 20 to 100%. This process yielded a total of five fractions (F1–F5). Thin-layer chromatography revealed distinct metabolite profiles under UV illumination. Antibacterial screening identified F5 as the most active fraction, as shown in Supplementary Figure S8, exhibiting broad-spectrum inhibition with zones of 25 mm (*Micrococcus luteus*), 27 mm (*Escherichia coli*), and 22 mm (*Staphylococcus aureus*) while no activity against *Bacillus thuringiensis* and *Enterococcus faecium*.

**Figure 11:**
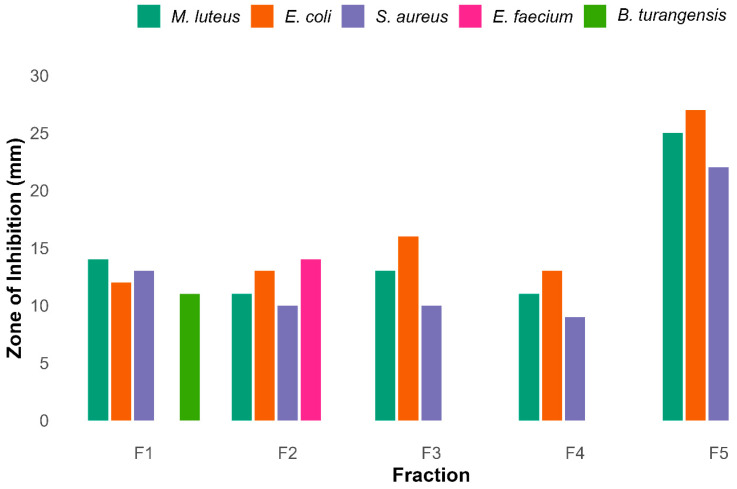
Antibacterial activity of five different rude fractions F1–F5 against diverse bacteria. Among these fractions F5 shows maximum inhibitory activity against 3 pathogenic bacteria, while F1 and F2 showed broader activity against bacteria.

Other fractions displayed narrower activity spectra. F1 and F2 exhibited moderate to selective inhibition, while F3 and F4 were primarily active against Gram-negative strains. DMSO served as a negative control. A detailed analysis of these observations is given in the bar graph in Figure 11.

To further analyze the fractions with pronounced antibacterial potential, F2 and F5 require further purification. Therefore, fractions F2 and F5 were further purified using Sephadex LH-20 column chromatography. Approximately 3 mL of the subfraction was collected and analyzed via TLC to combine similar pools of compounds and separate those with distinct spots on TLC plates. The compound fractions were further checked via HPLC, where necessary. The subfractions were prepared, and their antibacterial and antioxidant activity was checked. The bioactive fractions were further purified using reverse-phase HPLC to identify the sole compound purified.

### 3.13. QTOF-MS Analysis

**Figure 12:**
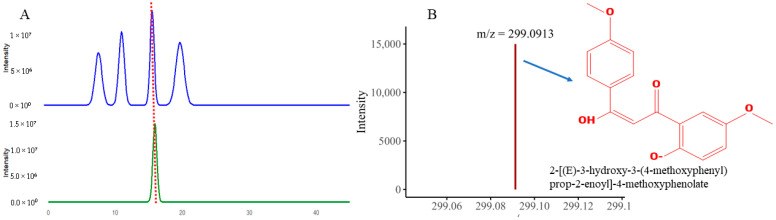
The figure revealed (**A**) HPLC analysis peak of the purified compound (**B**) QTOF-MS analysis showing *m*/*z* value and structure of the isolated compound.

For QTOF-MS analysis to identify bioactive compounds, a specialized software, Compass Data Analysis v4.5, was used to identify the compounds. The bioactivity-guided isolation revealed that F2 led to the isolation of a single compound with a light-orange color. The purity of the compound was checked using TLC as shown in TLC as shown in Supplementary Figure S9 and further confirmed via analytical HPLC, as shown in Figure 12A. The QTOF-MS analysis was performed. The QTOF-MS analysis of the pure compound isolated from the F2 fraction revealed a major ion with mass 299.0913 in [M + H] ion mode with a molecular formula of C_17_H_15_O_5_ Figure 12B. The compound has high accuracy, with a ppm error of 0.5, mSigma 11.1 and a confidence score of 100. The proposed structure of the compound was 2-[(E)-3-hydroxy-3-(4-methoxyphenyl) prop-2-enoyl]-4-methoxyphenolate; a conjugated aromatic molecule with an increased degree of saturation (RDB 10.5), directing to a polyphenolic or phenylpropanoid-like backbone. In short, these findings propose that the pronounced bioactivity of the F2 fractions can be attributed to this natural product.

**Figure 13:**
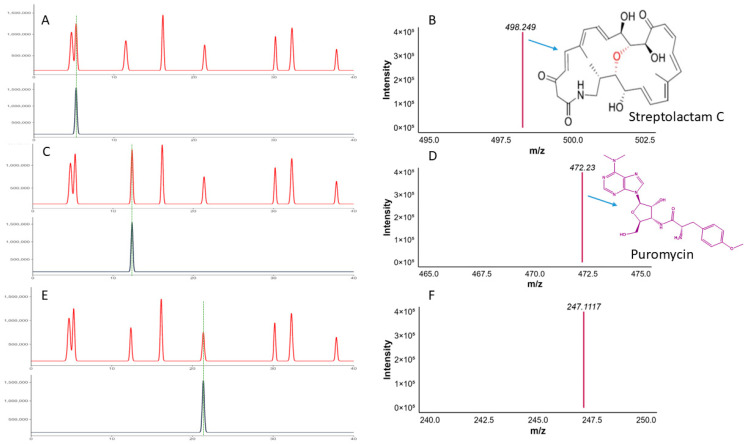
The Figure shows the (**A**,**B**) HPLC and QTOF-MS analysis of the pure compound Streptolactam C, (**C**,**D**) HPLC and QTOF-MS analysis of the puromycin, and (**E**,**F**) HPLC QTOF-MS analysis of the novel compound.

Similarly, the F5 fractionation resulted in the purification of three distinct peaks. The compound exhibits excellent antibacterial activity. The purity was verified by analytical HPLC, as shown in Figure 13A.

Among these, compound 1 has a mass of 498.2486 [M + H], conforming a molecular formula of C_28_H_36_NO_7_ (score of 100, ppm error of −1.7, and mSigma of 29.7). This compound matches Streptolactam C, a macrolactam antibiotic initially discovered in the deep-sea bacterium *Streptomyces*, supporting its identification based on accurate mass and known natural product data (Figure 13B).

Similarly, compound 2 with *m*/*z* 472.2300 in [M + H] ion mode, and proposed formula with high accuracy is C_22_H_30_N_7_O_5_ with (ppm error 0.6, score 100). MS/MS analysis showed characteristic fragment ions (i.e., *m*/*z* 235.1191, 251.1140), which are reliable indicators of aminonucleoside antibiotics. The HPLC analysis of the pure compound along with the MS/MS fragmentation features strongly suggest that the compound is Puromycin, formerly isolated from *Streptomyces alboniger* (Figure 13C,D).

Another compound 3 with *m*/*z* value of 247.1117 in ([M + H]^+^) matches the molecular formula of C_18_H_15_O, with a high RDB of (11.5), demonstrating a polycyclic aromatic structure. The mass correctness (−4 ppm error) and molecular features are consistent with those of bacterial polyketides, potentially related to angucyclinone or anthraquinone scaffolds. Although no exact match was found in the present databases, its profile suggests a possible novel bioactive compound (Figure 13E,F).

**Figure 14:**
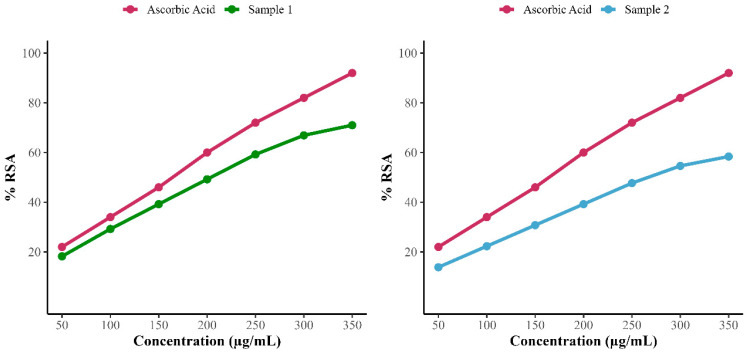
Antioxidant scavenging activity of 2-[(E)-3-hydroxy-3-(4-methoxyphenyl) prop-2-enoyl]-4-methoxyphenolate (sample 1) and F5 subfraction fraction including (Streptolactam C, puromycin, and putative aromatic polyketide (sample 2).

The antioxidant activity of these 2-[(E)-3-hydroxy-3-(4-methoxyphenyl) prop-2-enoyl]-4-methoxyphenolate from fraction F2, and Streptolactam C, Puromycin, and a putative aromatic polyketide isolated from F5 revealed 71% and 58.39% radical scavenging activity, as shown in Figure 14.

## 4. Discussion

Extremotolerant bacteria inhabiting harsh environments are increasingly recognized as valuable sources of novel bioactive secondary metabolites. In the current study, bacterial isolates from the extreme habitat of Chamalang Cave, Kohlu Balochistan, Pakistan, were assessed for their antimicrobial and antioxidant potential. Among them, isolate C1, identified as *Rhodococcus jialingiae*, demonstrated exceptional bioactivity.

C1 (*R. jialingiae*) exhibited broad-spectrum antibacterial activity against multidrug-resistant (MDR) strains like *Escherichia coli*, *Staphylococcus aureus*, and *Micrococcus luteus* with prominent inhibition zones of ~22–27 mm, equivalent to or exceeding standard antibiotics. These results suggest the presence of potent antibacterial compounds with therapeutic promise [34]. Unlike previously described *Rhodococcus* species that typically target closely related strains [35], our isolate displayed broader target specificity, reinforcing its potential clinical relevance.

The findings align with the growing evidence that extremophiles produce chemically diverse metabolites as adaptive mechanisms to survive hostile conditions [36]. Such compounds often possess unique scaffolds that can bypass conventional resistance pathways, making them attractive for drug development [36].

In addition to its antimicrobial properties, *R. jialingiae* exhibited significant antioxidant activity, with 71% and 58.39% DPPH radical scavenging value of two compounds comparable to or higher than those reported for other extremophile-derived actinobacteria [37]. These findings are also consistent with findings that pigments such as carotenoids or melanin in extremophiles serve dual roles in antioxidant defense and protective coloration [38]. Notably, the distinct signatures of *R. jialingiae* pigments suggest structural novelty, warranting further exploration for use as eco-friendly colorants in pharmaceuticals, cosmetics, or food applications [39].

A key highlight was the detection of a potential compound 2-[(E)-3-hydroxy-3-(4-methoxyphenyl) prop-2-enoyl]-4-methoxyphenolate from fraction F2, and Streptolactam C, puromycin, and a putative aromatic polyketide, a novel compound in the bioactive F5 fraction using QTOF-MS. Streptolactam C was previously isolated from deep-sea *Streptomyces* [40], while puromycin was isolated from *Streptomyces alboniggerin* 1952. This compound shows remarkable activity against bacteria, inhibiting protein synthesis.

The dominant molecule, with an *m*/*z* of 247.1117, has no known match in current databases. Its nitrogen-rich composition, comprising seven nitrogen atoms, points toward a putative aromatic polyketide, distinguishing it from common actinobacterial metabolites such as polyketides and terpenoids. Compared to the ~911 Da polyketide described by [35], this compound is smaller and chemically distinct. Although similar small molecules have occasionally been reported from extremotolerant bacteria, the observed formula and spectral data appeared to be novel. These observations highlight the importance of mining unconventional microbial taxa for novel chemistries. Complete structural elucidation via NMR or X-ray crystallography is necessary to confirm novelty and bioactive mechanisms [41], but the current data offers a compelling basis for further investigation.

Additionally, the current study signifies the role of cultivation conditions in maximizing the production of bioactive metabolites. *R. jialingiae* yielded the highest biomass under mesophilic, nutrient-rich conditions, at pH 7 and 30 °C, using peptone and maltose as nitrogen and carbon sources. Meanwhile, the extractable compounds were optimally produced at pH 7 and 30 °C, using peptone and glucose as nitrogen and carbon sources, respectively. Specifically, the addition of 1.0 g/L peptone and 0.5 g/L glucose significantly enhanced metabolite yields, as confirmed by two-way ANOVA. These findings revealed that the study isolate *R. jialingiae* exhibits the phenomenon of carbon catabolite repression (CCR) and nitrogen repression, which facilitates the increased production of secondary metabolites and their precursors in response to changing concentrations of favorable carbon and nitrogen sources. This phenomenon enables the production of primary metabolites, which in turn support cellular processes and growth. In the presence of a high concentration of maltose and glucose as C sources and increased concentration of peptone as a N source, the CCR and nitrogen repression block the expression of gene clusters responsible for secondary metabolites, but when these sources (glucose and nitrogen) are depleted, the CCR is depressed and this makes a way for the other biosynthetic gene clusters to be transcribed and translated, similar to our finding [42,43]. These findings also pave the way for genetic engineering and molecular approaches to enhance the biosynthetic potential of *R. jialingiae* C1, leading to improved production of bioactive compounds. Extremotolerant organisms are adapted to extreme environments with various environmental and nutritional limiting factors, which leads to the increased expression of genes responsible for secondary metabolites. However, laboratory conditions may or may not trigger the full expression of their biosynthetic pathways, which are critical for fermentation, scale-up, and downstream applications. Therefore, it is essential to determine the mechanisms of gene expression that lead to the production of secondary and valuable metabolites.

In short, *R. jialingiae* demonstrates the biotechnological potential of extremotolerant bacteria, and presents broad-spectrum antimicrobial activity, potent antioxidants, and structurally unique metabolites. These findings support continued exploration of extremophiles as promising sources for novel therapeutics.

## 5. Conclusions

The current study reveals the previously unexploited potential of extremophilic bacteria for the discovery of bioactive natural products. Among the strains isolated, the C1 *Rhodococcus jialingiae* isolated from the cave showed broad-spectrum activity against pathogenic bacteria. Detailed cultural conditions optimization, including media composition, pH, temperature, and nutrient sources, as well as concentration, specifically improved both biomass and extractable compound production. Bioassay-guided purification led to the identification of 2-[(E)-3-hydroxy-3-(4-methoxyphenyl) prop-2-enoyl]-4-methoxyphenolate from fraction F2. Additionally, Streptolactam C, puromycin, and a putative aromatic polyketide with strong antibacterial activity were identified from Fraction F5. These results not only underscore the biosynthetic capabilities of environmental *Rhodococcus* strains but also highlight their significance in natural product discovery pipelines for addressing antimicrobial resistance.

## Data Availability

The original contributions presented in this study are included in the article. Further inquiries can be directed to the corresponding author.

## Supplementary Materials

The following supporting information can be downloaded at https://www.mdpi.com/article/10.3390/biom15081071/s1: Figure S1: The figure shows the colony growth patterns, surface morphology, colony size, and color of six isolates on Agar plate. Among these the C1 isolate was chosen for further study; Figure S2: Antibacterial activity of the C1 isolate against the selected test organisms; Figure S3: Biochemical characterization of *Rhodococcus jialingiae*, showing negative results for (A) Tween 80 lipid hydrolysis, (B) gelatin liquefaction, and (C) amylase test; Figure S4: Correlation between bacterial biomass and extractable compound concentration across four media; Figure S5: Correlation between biomass and extractable compound levels; Figure S6: Smoothed trends of biomass and metabolite production across pH; Figure S7: Correlation between biomass and extractable compound levels; Figure S8: TLC analysis of the 5 different fractions. and antibacterial activity of these fractions against diverse pathogenic bacteria; Figure S9: TLC analysis of 2-[(E)-3-hydroxy-3-(4-methoxyphenyl) prop-2-enoyl]-4-methoxyphenolate isolated from Fraction 2; Table S1: Parameter for secondary metabolites production; Table S2: Composition and culture conditions of the optimized medium (C2-OSM) used for large-scale fermentation of *Rhodococcus jialingiae* C1; Table S3: The Morphologic and cultural features of the nominated isolates; Table S4: Biochemical properties of *Rhodococcus* species based on their observed enzymatic activities; Table S5: Two-way ANOVA analysis of biomass yield and extractable metabolite production; Table S6: Two-way ANOVA analysis of biomass yield and extractable metabolite production.
