# Supplementary material for: Optimization and Characterization of Bioactive Metabolites from Cave-Derived Rhodococcus jialingiae C1
Source: Biomolecules. 2025 Jul 24;15(8):1071. doi: 10.3390/biom15081071 (PMC12383431; doi:10.3390/biom15081071)
Supplement: Supplementary file 1 [file biomolecules-15-01071-s001.zip › biomolecules-3761537-supplementary.pdf]

Optimization and Characterization of Bioactive Metabolites from Cave-Derived *Rhodococcus jialingiae C1*

Supplementary Data.

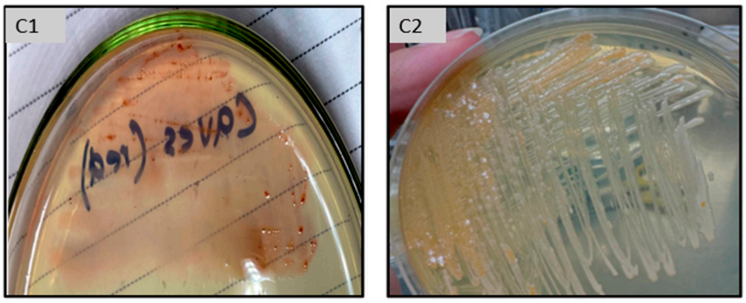

Supplementary Figure S1. The figure shows the colony growth patterns, surface morphology, colony size and color of six isolates on Agar plate. Among these the C1 isolate was chosen for further study.

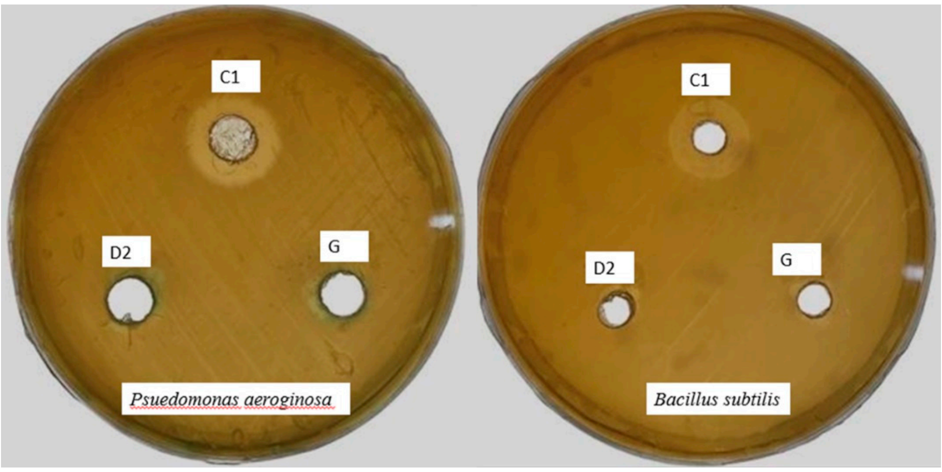

Supplementary Figure S2. Antibacterial activity of the C1 isolate against the selected test organisms.

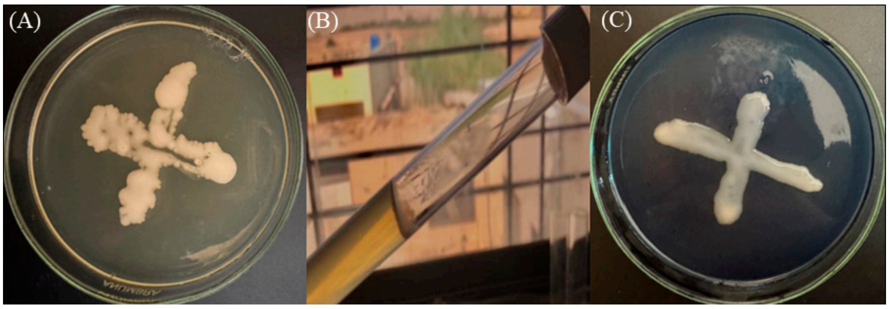

Supplementary Figure S3. Biochemical characterization of Rhodococcus jialingiae, showing negative results for (A) Tween 80 lipid hydrolysis, (B) gelatin liquefaction, and (C) amylase test.

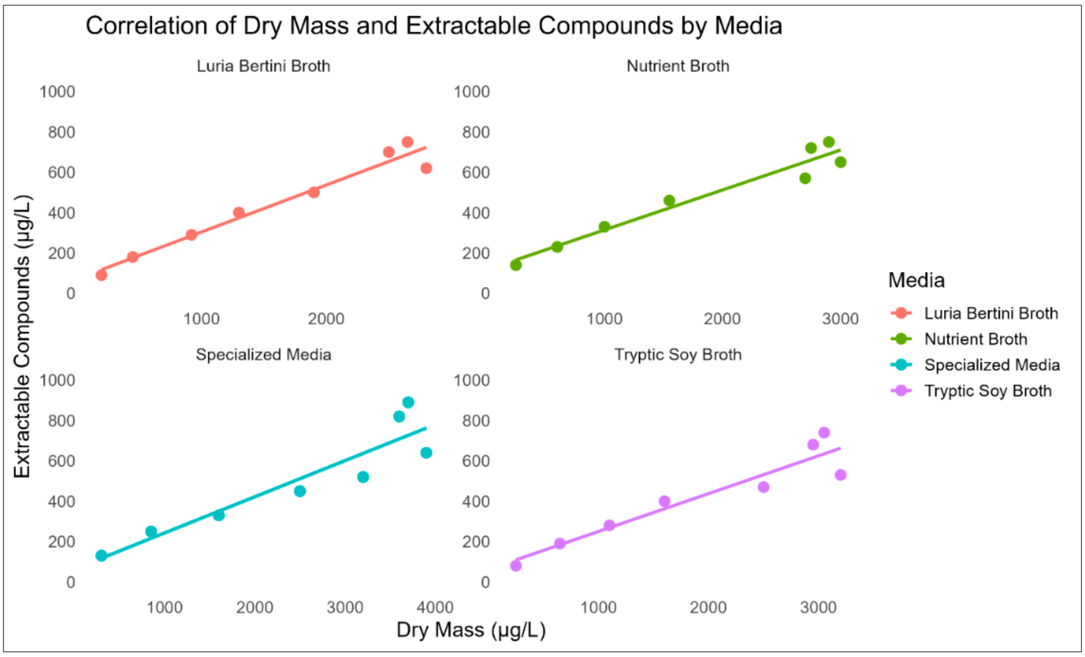

Supplementary Figure S4. Correlation between bacterial biomass and extractable compound concentration across four media.

Scatter plots (n = 8 per medium) with regression lines show strong positive correlations (Pearson's r = 0.97–0.99). These results highlight a consistent relationship between biomass and metabolite yield, supporting the use of biomass as a proxy for production optimization.

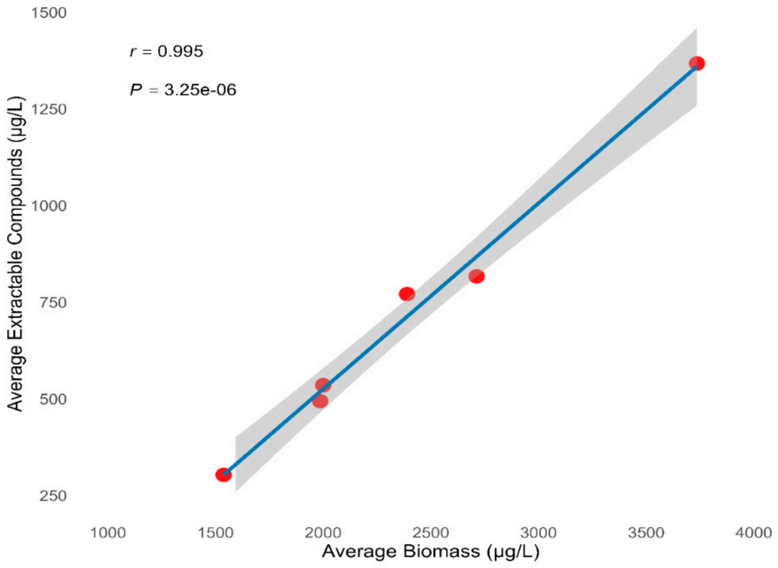

.

Supplementary Figure S5. Correlation between biomass and extractable compound levels.

Scatter plot illustrating a strong positive relationship between biomass and extractable yield across pH conditions (Pearson's r *= 0.995,* P *= 3.2 × 10^{-6}). The linear trend suggests metabolite output scales closely with biomass*

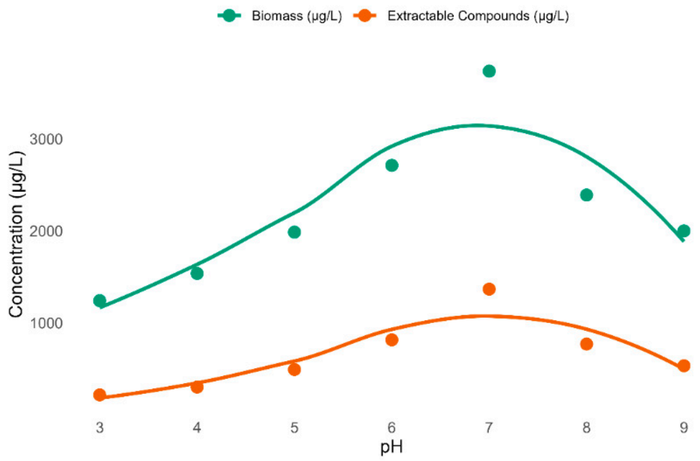

Supplementary Figure S6. Smoothed trends of biomass and metabolite production across pH.

Loess curves showing the response of biomass and extractable compounds across pH values. Both curves peak at pH 7, reflecting the coordinated influence of pH on microbial growth and secondary metabolism.

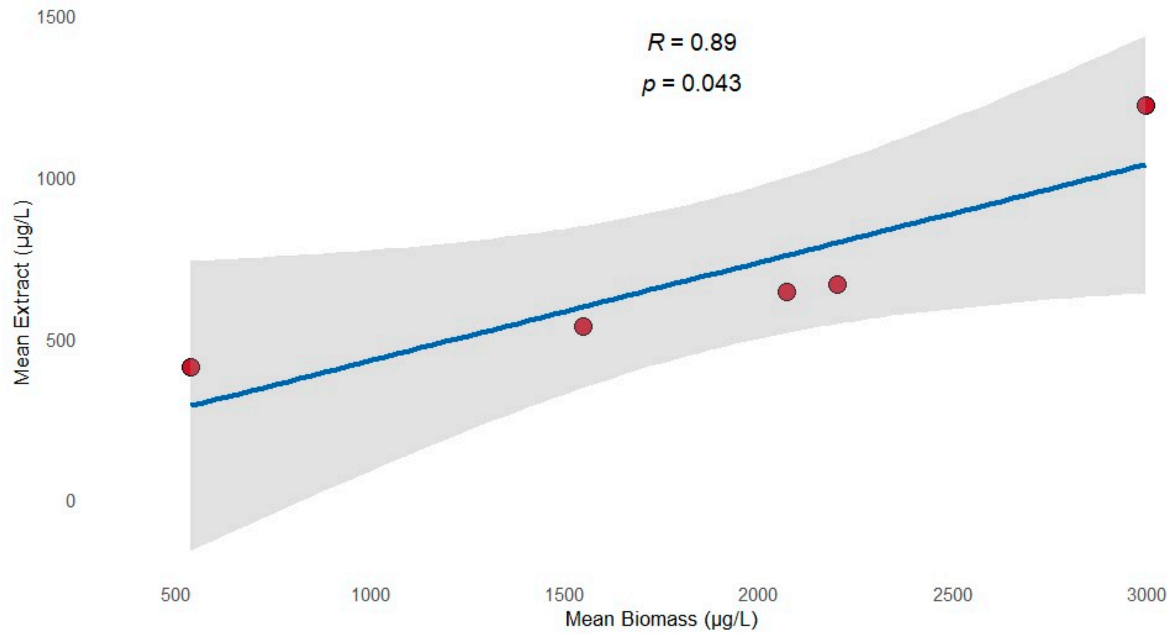

Supplementary Figure S7. Correlation between biomass and extractable compound levels.

Scatter plot demonstrates a robust positive association between biomass and extractable yield at Temperature conditions (Pearson's r *= 0.0.89,* P *= 0.043). The linear trend suggests metabolite output scales closely with biomass*

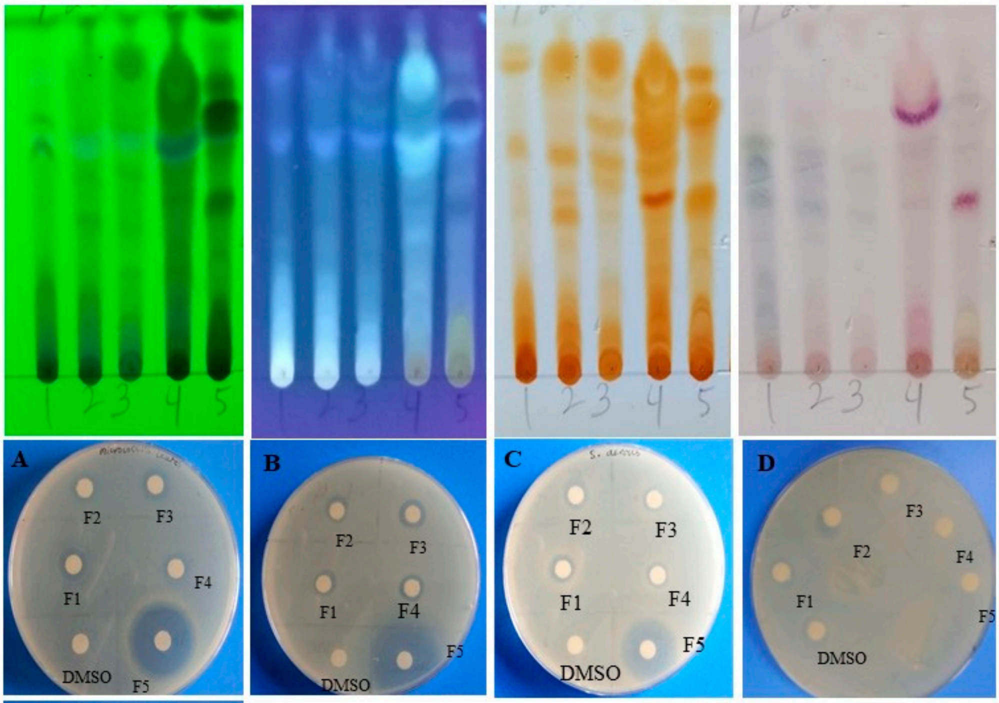

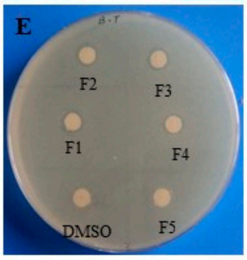

A. Micrococcus luteus
B. Escherichia coli
C. Staphylococcus aureus
D. Enterococcus faecium
E. Bacillus thuringiensis

Supplementary Figure S8. TLC analysis of the 5 different fractions. and antibacterial activity of these fractions against diverse pathogenic bacteria.

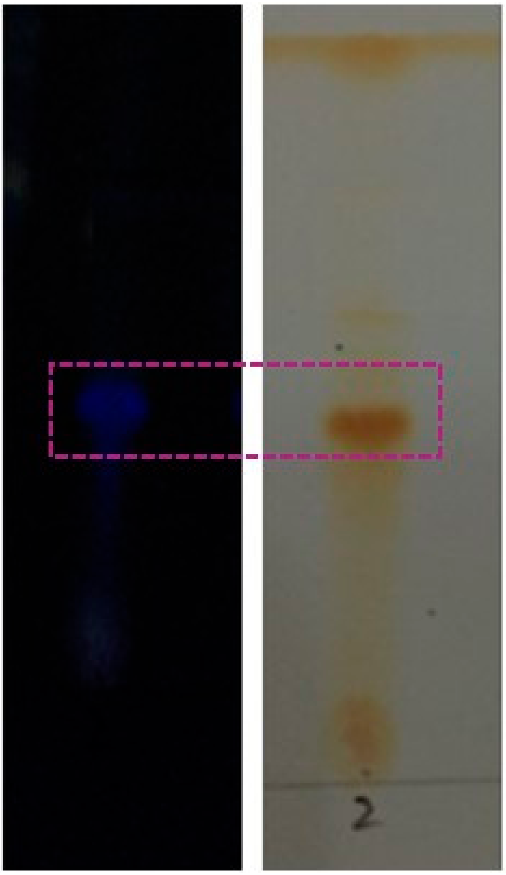

Supplementary Figure S9. TLC analysis of 2-[(E)-3-hydroxy-3-(4-methoxyphenyl) prop-2-enoyl]-4-methoxyphenolate isolated from Fraction 2

Supplementary Tables

Supplementary Table S1. Parameter for Secondary metabolites production.

Parameter	Conditions
Media	LB, Nutrient Agar, Tryptic Soy Agar, Specialized Media
Timepoints	192 hrs, sampled every 24 hours
Temperature (°C)	10, 15, 20, 25, 30, 37
pH	3-9
Nitrogen Source	Ammonium sulfate, Peptone, Yeast extract, Sodium nitrate (0.5–2 g/L)
Carbon Source	Glucose, Maltose, Sucrose, Starch (0.5-2 g/L)

Supplementary Table S2. Composition and culture conditions of the optimized medium (C2-OSM) used for large-scale fermentation of Rhodococcus jialingiae C1. *Medium components and cultivation parameters were selected based on systematic optimization of nutrient sources, pH, temperature, and incubation time to enhance biomass accumulation and secondary metabolite yield.*

Component	Concentration	Function
Peptone	1.0 g/L	Nitrogen source; enhances metabolite yield
Maltose	0.5 g/L	Carbon source; promotes biomass production
Yeast Extract	2.0 g/L	Provides growth factors and vitamins
K_2HPO_4	1.0 g/L	pH buffering and phosphate supply
$MgSO_4 \cdot 7H_2O$	0.5 g/L	Cofactor for enzyme function
NaCl	0.5 g/L	Maintains osmotic balance
pH	Adjusted to 7.0	Optimal for growth and metabolite production
Temperature	30 °C	Optimal for biomass and extract yield
Agitation	180 rpm	Ensures aeration and homogeneity
Incubation Time	168 hours	Peak for secondary metabolite production

Supplementary Table S3. The Morphologic and cultural features of the nominated isolates. The table describes the colony color, size, texture, elevation and gram reactions recorded during the primary screening. All isolates were selected with distinct morphology and antibiotic production

Isolates	Colony Morphology						Gram reaction
	Color	Size	Form	Texture	Opacity	Elevation	
C1	Red orange	Small	Circular	Moist/Shiny	Translucent	Flat	G +ve
C2	Light brown	Small	Circular	Moist/Shiny	Opaque	Convex	G +ve

Supplementary Table S4. Biochemical properties of Rhodococcus species based on their observed enzymatic activities.

Species	Tween-80 lipid hydrolysis	Gelatin liquification	Amylase production
Rhodococcus jialingiae.	Negative	Negative	Negative
Rhodococcus erythropolis	Strong positive	Positive	Positive
Rhodococcus qingshengii	Weakly positive	Weakly positive	Weakly positive

Supplementary Table S5. Two-way ANOVA analysis of biomass yield and extractable metabolite production.

The table summarizes the effects of Nitrogen source, concentration, and their interaction on microbial biomass and extractable metabolites. Bold values represent statistically significant differences (p < 0.001). Data are based on three biological replicates per condition.

Effect	DF	Sum Sq	Mean Sq	F	p-value
**Biomass Yield (µg/L)**					
Nitrogen source	3	1577951.6	525983.9	182.5	*<0.001*
Concentration	2	2932434.7	1466217.3	508.7	*<0.001*
Interaction	6	2390854.4	398475.7	138.2	*<0.001*
**Extractable Metabolites (µg/L)**					
Nitrogen source	3	244742.3	81580.8	57.9	*<0.001*
Concentration	2	513361.2	256680.6	182.3	*<0.001*
Interaction	6	1398618.6	233103.1	165.5	*<0.001*

Supplementary Table S6. Two-way ANOVA analysis of biomass yield and extractable metabolite production. The table summarizes the effects of carbon source, concentration, and their interaction on microbial biomass and extractable metabolites. Bold values represent statistically significant differences ($p < 0.05$). Data are based on three biological replicates per condition.

Effect	DF	Sum Sq	Mean Sq	F	p-value
**Biomass Yield (µg/L)**					
Carbon source	3	872401.0	290800.3	11.5	<0.001
Concentration	2	5278264.1	2639132.0	104.2	<0.001
Interaction	6	704588.2	117431.4	4.6	0.003
**Extractable Metabolites (µg/L)**					
Carbon source	3	661649.4	220549.8	160.2	<0.001
Concentration	2	536537.2	268268.6	194.8	<0.001
Interaction	6	617903.7	102984.0	74.8	<0.001
